# Case Report: IgG4-related ophthalmic disease presenting with unilateral proptosis

**DOI:** 10.3389/fimmu.2025.1652730

**Published:** 2025-10-27

**Authors:** Xiaodong Li, Xuewei Qin, Mei Chen, Xi Zheng

**Affiliations:** ^1^ The First Affiliated Hospital of Guizhou University of Traditional Chinese Medicine, Ophthalmology, Guiyang, China; ^2^ Traditional Chinese Medicine Hospital of Meishan, Ophthalmology, Meishan, China

**Keywords:** IgG4-related eye disease, unilateral orbital mass, glucocorticoids, case report, IgG4-ROD

## Abstract

IgG4-related eye disease (IgG4-ROD) is characterized by diffuse swelling or well-defined masses in the lacrimal glands, extraocular muscles, and eyelids, often accompanied by eyelid swelling, proptosis, and diplopia. It typically affects both sides symmetrically and can mimic various infectious, inflammatory, and neoplastic diseases. Early recognition and steroid therapy can significantly improve symptoms, but recurrence is common, necessitating careful and long-term follow-up. B-cell depletion therapy, such as rituximab, is increasingly being incorporated into standard treatment protocols. This article reports a case of IgG4-ROD misdiagnosed as a unilateral orbital mass, summarizing its clinical features, diagnostic approach, and treatment outcomes. Combined with a literature review and analysis, this study aims to provide reference for the clinical identification of IgG4-ROD.

## Introduction

IgG4-related disease (IgG4-RD) is a chronic autoimmune disease characterized by elevated serum IgG4 levels, IgG4-positive plasma cell infiltration in tissues, reticular fibrosis, and occlusive venulitis. The etiology remains unclear, and it can affect multiple organs throughout the body (1). When multiple ocular structures, including the lacrimal glands, extraocular muscles, eyelids, and trigeminal nerve, are involved and ocular symptoms are present, the condition is referred to as IgG4-related ocular disease (IgG4-ROD) (2). Studies (3) have shown that IgG4-ROD may also involve facial structures, salivary glands, lymph nodes, and the mucosa of all paranasal sinuses. Previous clinical diagnosis of IgG4-ROD has generally followed the guidelines recommended by a Japanese research group (4). Since the diagnosis and treatment of IgG4-ROD require multidisciplinary collaboration, the Chinese Medical Association has developed the latest expert consensus on the diagnosis and treatment of IgG4-ROD based on a summary of domestic and international clinical research results and diagnostic and therapeutic experiences. This consensus emphasizes that the diagnosis of IgG4-ROD should be based on a comprehensive evaluation of ocular imaging, serum IgG4 levels, ocular tissue pathology, and systemic examination results, and it recommends the indications and methods for drug therapy with glucocorticoids, immunosuppressants, and biologics, as well as surgical treatment (5). Since IgG4-ROD can mimic various infectious, inflammatory, and neoplastic diseases, it is particularly prone to misdiagnosis in clinical practice as lacrimal gland tumors, inflammatory pseudotumors, and other orbital space-occupying lesions.

We report a case of IgG4-ROD presenting with unilateral proptosis and decreased visual acuity as initial symptoms, which was clinically misdiagnosed as optic nerve tumor. This report aims to enhance clinical awareness and diagnosis of IgG4-ROD, increase understanding of the disease, and reduce misdiagnosis and missed diagnosis. A review of the literature reveals that similar cases are rarely reported.

## Case presentation

One year prior to presentation, the patient developed right eye proptosis, periorbital edema, decreased visual acuity (VA), blurred vision, and ocular pruritus without identifiable precipitating factors. He denied photophobia, epiphora, conjunctival injection, or orbital/ocular pain. He initially sought care at a local hospital, where he received empiric treatment with unspecified antibiotic eye drops, which failed to alleviate symptoms. Thereafter, the symptoms progressively deteriorated despite treatment. He was subsequently referred to our ophthalmology clinic, where a provisional diagnosis of “right orbital space-occupying lesion” was made based on clinical and imaging findings. After further examinations, elective surgery was scheduled. The patient denied a history of hypertension, diabetes, coronary heart disease, or infectious diseases. Five years prior, he underwent surgical drainage for a pulmonary abscess at a local hospital, with an uneventful postoperative course. He has a 40-year history of alcohol consumption (current daily intake not specified) and a 40-year smoking history (20 cigarettes/day), with abstinence from smoking for the past five years.

### Ophthalmic examination

**Figure 1:**
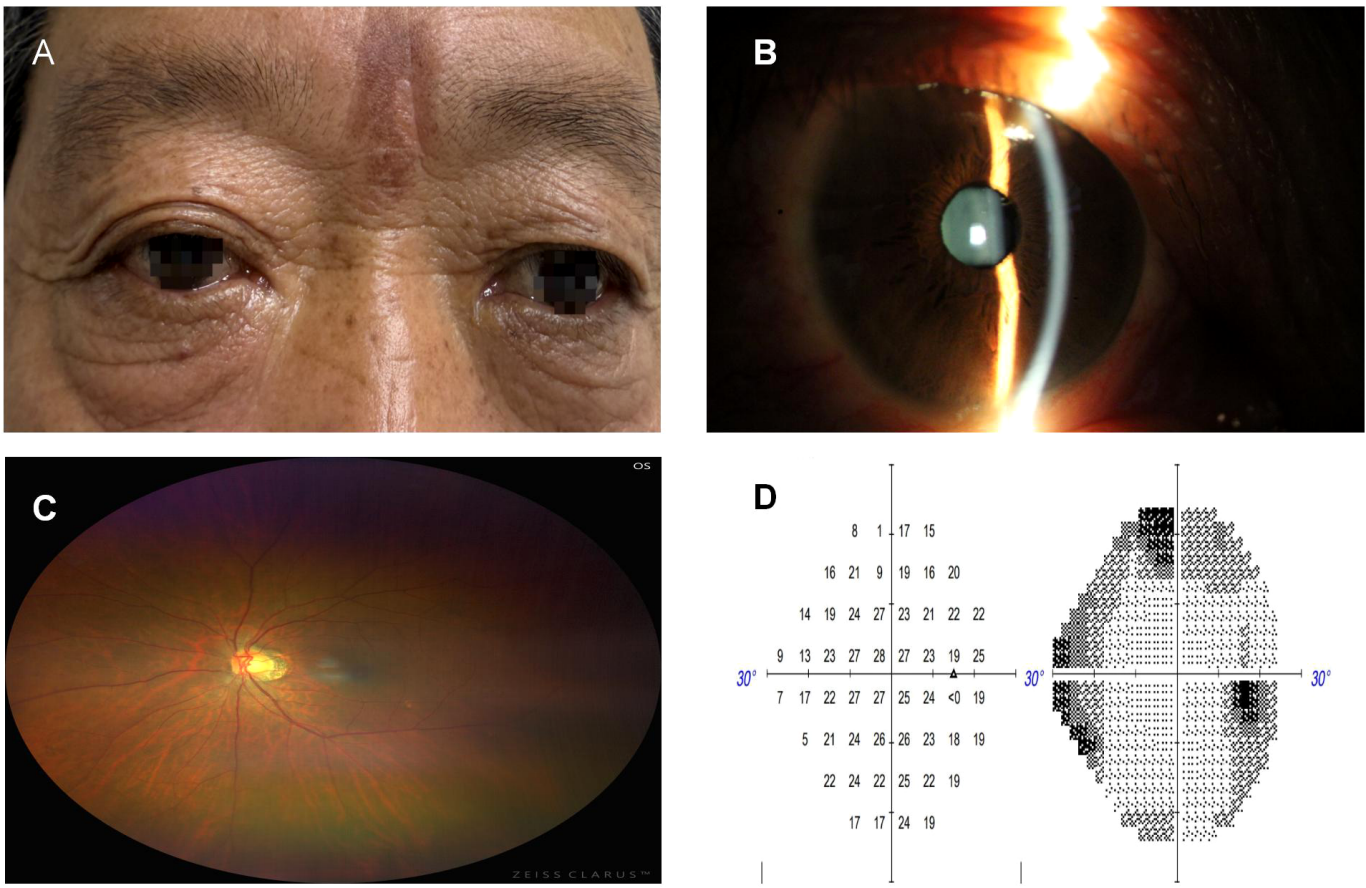
**(A)** Photograph of both eyes showing marked protrusion of the right eye; **(B)** Slit-lamp photograph of the right eye showing conjunctival hyperemia, transparent cornea, and cloudy lens; **(C)** Fundus photograph of the right eye showing peripapillary atrophy and leopard-spot fundus changes; **(D)** Dynamic visual field of the right eye showing enlarged physiological blind spot and peripheral scotoma.

Visual acuity: right eye 0.1, left eye 0.5, marked proptosis of the right eye, conjunctival hyperemia; cornea transparent, adequate anterior chamber depth, Tyn (-), pupils round (diameter 3 mm), sensitive to light reflex; lens and vitreous opacity; fundus shows leopard-spot pattern. Eye movements in all directions are unrestricted; intraocular pressure: right eye 17 mmHg, left eye 16 mmHg. Dynamic visual field examination: enlarged physiological blind spot in the right eye, peripheral visual field loss, reduced visual acuity. As shown in **Figure 1**.

### Additional tests

**Figure 2:**
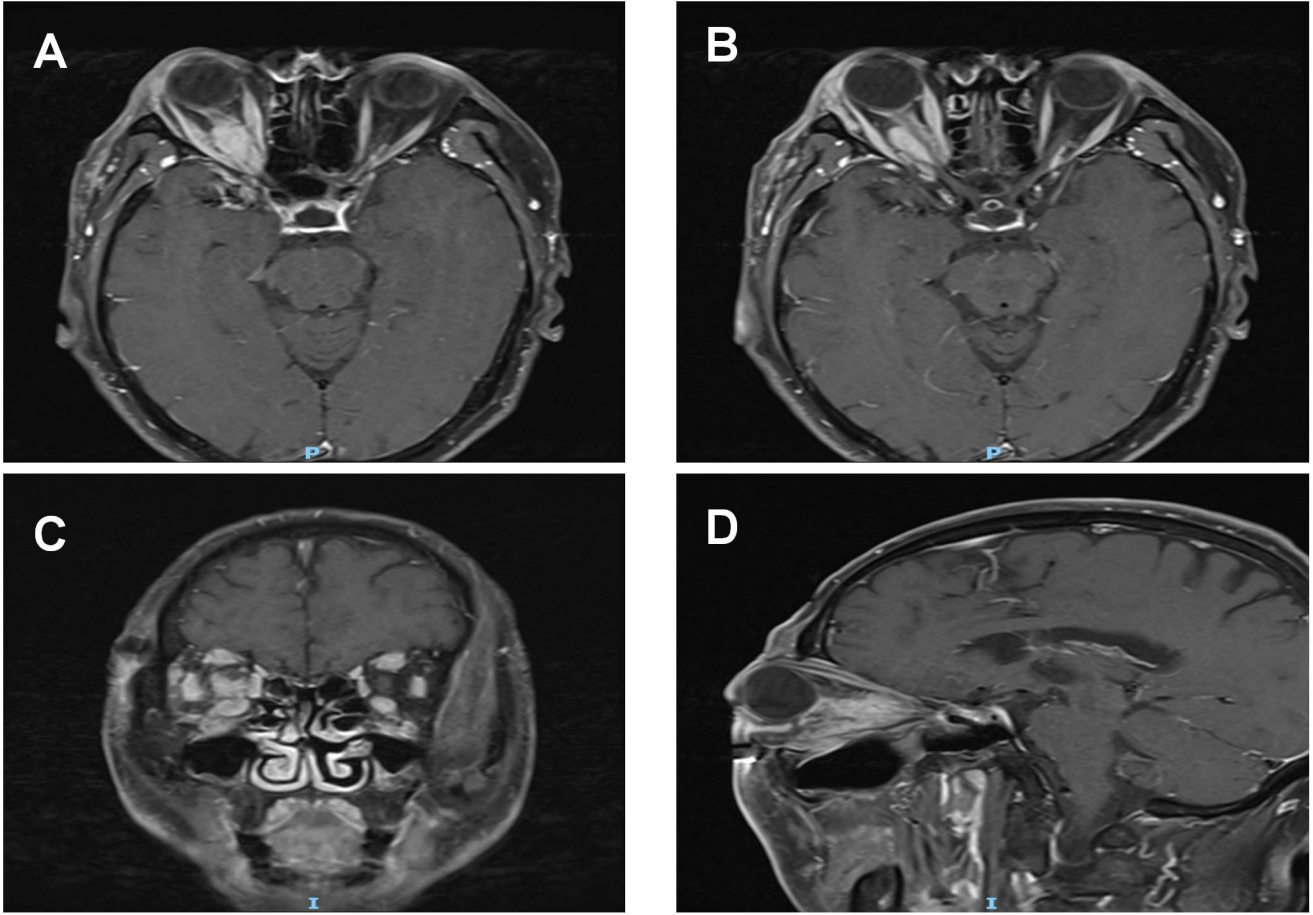
Pre-treatment MRI orbital plain scan + contrast enhancement: **(A, B)** A spindle-shaped soft tissue mass (long diameter: 20 mm × short diameter: 12 mm) is visible from the intracanalicular segment to the intraorbital segment of the right optic nerve. On T1WI, the mass exhibits isointense to hypointense signals with ill-defined borders; it surrounds the optic nerve without invading the optic nerve parenchyma, and no optic nerve transection or eccentric compression is observed. The bellies of the right medial rectus and lateral rectus muscles show symmetric thickening, presenting isointense signals on T1WI. Their tendon insertion sites are clear, and moderate homogeneous enhancement is seen on contrast-enhanced scan, with no "swelling at the myotendinous junction".The right lacrimal gland displays diffuse enlargement, showing isointense signals on T1WI and homogeneous enhancement after contrast administration. **(C, D)** T1WI coronal and sagittal images, respectively.The mass, thickened extraocular muscles, and lacrimal gland all present moderate homogeneous enhancement, with an enhancement degree lower than that of blood vessels. No "ring enhancement" or "necrotic area" is noted. The mucosa of the bilateral maxillary sinuses and ethmoid sinuses is thickened with mild enhancement.

1. Key laboratory positive indicators: Immunoglobulin: IgG 18.84 g/L elevated, IgA 0.89 g/L decreased; IgG4 level: 7.72 g/L, significantly elevated; Thyroid function: TSH 5.480 μIU/mL elevated, thyroid antibodies negative; Other: Tumor markers, tuberculosis-related tests (PPD, T-SPOT.TB) were all negative. Imaging features: Optical coherence tomography (optic disc): Average thickness of the nerve fiber layer of the optic disc in the right eye was 72 μm, and in the left eye was 90 μm. Enhanced MRI of the orbit: right eye protrusion (right side 23 mm, left side 19 mm), thickening of the right eye’s medial and lateral rectus muscles, a slightly elongated T1/T2 signal shadow in the right optic nerve tract, approximately 20 mm × 12 mm, with unclear boundaries from the optic nerve, slight enlargement of the right lacrimal gland, and blurred orbital fat with fibrous exudation; The lesion shows significant enhancement after contrast administration. Thickening of the mucosa of both maxillary sinuses and ethmoid sinuses is noted. As shown in **Figure 2**. Chest CT: Bronchiectasis and chronic inflammation of the right upper lobe of the lung, multiple nodules in both lungs (maximum 4 mm × 3 mm). These nodules were considered unrelated to the previous pulmonary abscess (5 years prior, treated with surgical drainage and uneventful recovery), as they showed no contiguous involvement with the prior abscess site and lacked features of active infection (e.g., cavitation, surrounding consolidation). Atherosclerosis of the aorta and coronary arteries, hepatic calcifications, and splenic capsule calcification were also noted.

### Clinical diagnosis

Based on the aforementioned examination results, after a multidisciplinary consultation involving the Rheumatology and Immunology Department, Endocrinology Department, and Respiratory Department, the following diagnoses were proposed: 1. Right eye IgG4-related eye disease (IgG4-ROD) 2. Bilateral age-related cataracts 3. Bilateral maxillary sinusitis and ethmoid sinusitis 4. Subclinical hypothyroidism 5. Right lung bronchiectasis with infection 6. Bilateral pulmonary multiple nodules.

### Treatment plan

Since the patient and family refused a biopsy of the optic nerve tumor, the following treatment was administered: intravenous infusion of methylprednisolone sodium succinate 1000 mg/3 days + 500 mg/3 days + 250 mg/3 days, Subsequently, the treatment was changed to oral prednisolone acetate 60 mg/day, combined with medications to protect the gastric mucosa, supplement calcium, and prevent hypokalemia, along with oral methylcobalamin tablets for neuroprotective therapy.

### Treatment outcomes

**Figure 3:**
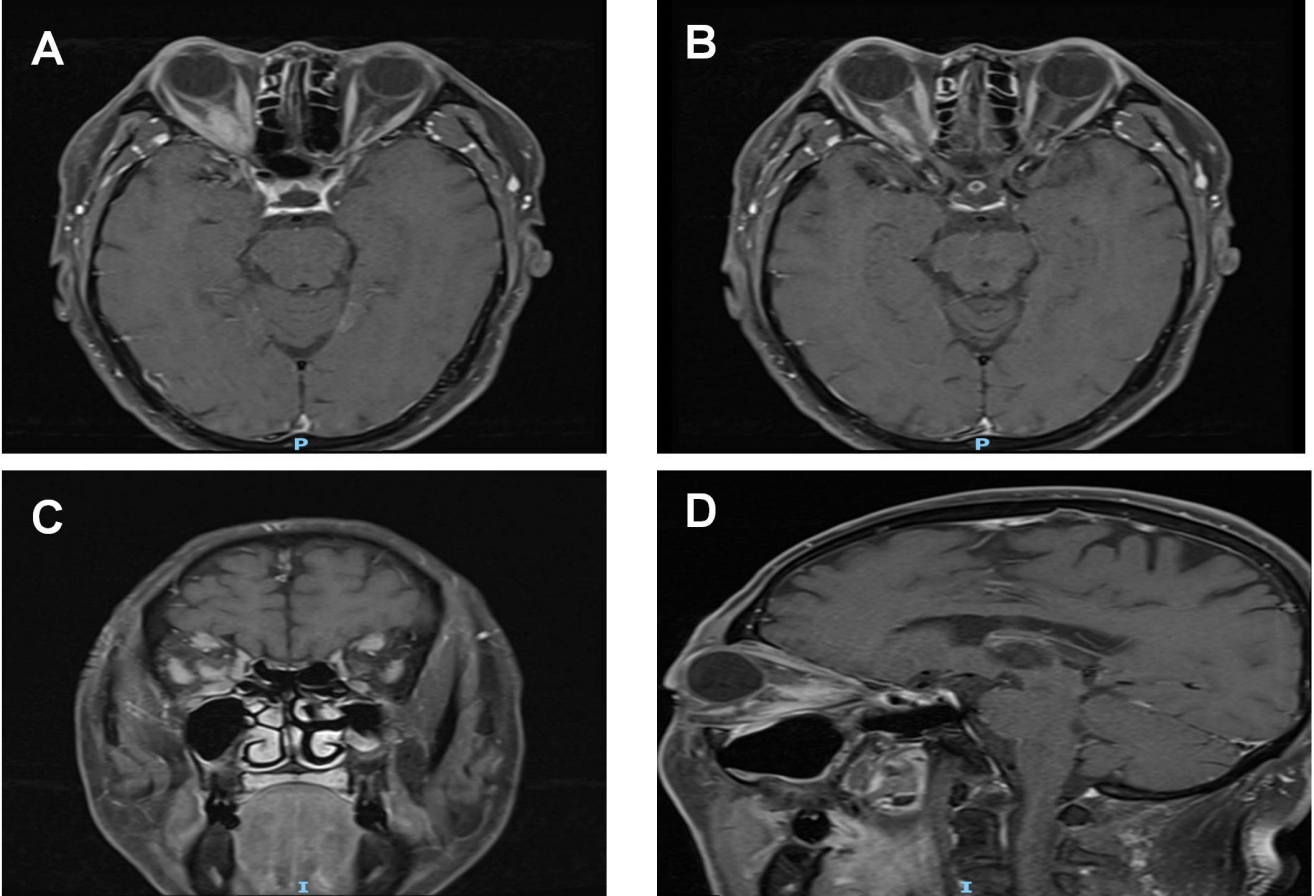
Post-treatment MRI orbital plain scan + contrast enhancement: **(A, B)** The mass around the right optic nerve has shrunk to 16 mm × 9 mm (a 20% reduction compared to pre-treatment), with clearer borders than before and a distinct boundary from the optic nerve. The thickness of the right medial rectus and lateral rectus muscles has decreased to 4 mm (approaching the normal range), the volume of the lacrimal gland has shrunk to 14 mm, and the degree of enhancement has weakened after contrast administration. **(C, D)** T1WI coronal and sagittal images, respectively.

After two weeks of treatment, the patient’s right eye protrusion improved significantly, with an eye protrusion of 19 mm, no eye swelling or pain, and improved right eye vision to 0.25. A follow-up orbital MRI showed a slightly elongated T1/T2 signal shadow in the right optic nerve tract, approximately 16 mm × 9 mm, and a reduction in the volume of the right lacrimal gland compared to previous measurements; as shown in **Figure 3**. Serum IgG4 levels decreased to 5.56 g/L upon follow-up. The patient requested outpatient follow-up treatment after discharge, with oral steroid dosage reduced by 5 mg weekly. One month later, serum IgG4 levels decreased to 3.35 g/L upon follow-up. Outpatient follow-up showed that the IgG4 level was 3.65 g/L on June 13, 2025, and 3.31 g/L on August 18, 2025.

To clearly demonstrate the patient’s disease progression and treatment response, the key time points, examination results, and intervention measures are summarized in **Table 1**.

**Table 1:** Summary table of diagnosis and treatment timeline for IgG4-ROD case.

TimeNode	KeyEvents
1 year before onset	Developed right eye protrusion, eye swelling, decreased vision (right eye 0.1), blurred vision, and eye itching without obvious precipitating factor. No photophobia, tearing, redness, or eye pain.
After onset (local hospital visit)	Visited a local hospital and was treated with “a certain antibiotic eye drop,” but symptoms worsened gradually.
At admission (our hospital)	Ophthalmic examination: Right eye proptosis (23mm), conjunctival hyperemia, lens opacity, leopard-spot fundus changes; dynamic visual field showed enlarged physiological blind spot and peripheral scotoma. Laboratory tests: Serum IgG4 7.72g/L (significantly elevated), IgG 18.84g/L (elevated), TSH 5.480μIU/mL (elevated); tumor markers and tuberculosis-related tests negative. Imaging: Enhanced orbital MRI revealed right eye protrusion, thickening of medial and lateral rectus muscles, a 20mm×12mm slightly elongated T1/T2 signal shadow in the right optic nerve tract (unclear boundary), mild enlargement of the lacrimal gland, and blurred orbital fat with fibrous exudation. Preliminary diagnosis: Right orbital mass (scheduled for surgery).
After multidisciplinary consultation	Clinical diagnosis: 1. Right eye IgG4-related ophthalmic disease (IgG4-ROD); 2. Bilateral age-related cataracts; 3. Bilateral maxillary sinusitis and ethmoid sinusitis; 4. Subclinical hypothyroidism; 5. Right lung bronchiectasis with infection; 6. Bilateral pulmonary multiple nodules.
Start of treatment	Patient refused optic nerve tumor biopsy. Treatment included intravenous methylprednisolone (1000mg/day for 3 days → 500mg/day for 3 days → 250mg/day for 3 days), followed by oral prednisolone acetate 60mg/day, combined with gastric mucosa protectants, calcium supplements, potassium supplementation, and methylcobalamin for neuroprotection.
2 weeks after treatment	Efficacy: Significant improvement in right eye protrusion (19mm), resolution of eye swelling, visual acuity improved to 0.2. Laboratory tests: Serum IgG4 decreased to 5.56g/L. Imaging re-examination: Orbital MRI showed reduced lesion size in the right optic nerve tract (16mm×9mm), decreased lacrimal gland volume, and alleviated extraocular muscle thickening.
1 month after treatment (follow-up)	Laboratory tests: Serum IgG4 further decreased to 3.35g/L. Treatment adjustment: Oral steroid dosage reduced by 5mg weekly, with outpatient follow-up.

## Discussion

IgG4-RD is a rare fibrotic inflammatory disease affecting multiple organs that was first recognized after 2003. Its pathogenesis is complex, involving genetic and environmental factors, with immune responses typically mediating organ damage and promoting fibrosis, a key feature of the disease (6). Although IgG4-RD has clinical and histological characteristics, the lack of validated diagnostic criteria often makes diagnosis challenging, requiring a multidimensional approach integrating clinical, radiological, and serological data (7). Among these, IgG4-ROD as an ocular complication is frequently misdiagnosed or overlooked in clinical practice. The latest diagnostic criteria for IgG4-ROD (5) are as follows: (1) Imaging studies showing enlargement of the lacrimal gland, trigeminal nerve, or extraocular muscles, various ocular masses, proliferative lesions, or diffuse sinus inflammation; (2) Elevated serum IgG4 levels (≥1.35 g/L); (3) Histopathological examination showing lymphocytic and plasma cell infiltration, sometimes with fibrosis, and often with germinal centers. When all three criteria are met, IgG4-ROD can be diagnosed. Notably, it is important to distinguish between diagnostic criteria and classification criteria in clinical practice. Diagnostic criteria (e.g., 2024 expert consensus (5)) are used for clinical confirmation, requiring integration of clinical, imaging, serological, and histopathological findings. Classification criteria, by contrast, are primarily for research purposes to ensure uniformity in study populations, and may include stricter thresholds for IgG4+ plasma cell counts or fibrosis.

This case presents with unilateral proptosis and decreased visual acuity as the primary initial symptoms. Combined with enhanced MRI of the orbit showing “a fusiform soft tissue mass enveloping the optic nerve tract,” “thickening of the extraocular muscles,” and “enlargement of the lacrimal gland,” the following possibilities were considered at the time of admission: (1) Right optic nerve tumor, such as optic nerve glioma or meningioma, as orbital imaging suggests an occupying lesion in the optic nerve region, necessitating differentiation from optic nerve tumors. However, laboratory tests upon admission showed normal tumor markers, MRI did not reveal bone destruction, the mass did not enhance after contrast administration, and the optic nerve mass significantly shrunk after steroid therapy, which does not align with tumor biology. Therefore, the diagnosis of optic nerve tumor in the right eye was temporarily ruled out. IgG4-related disease (IgG4-ROD) involving the optic nerve can cause optic nerve damage. Optic nerve damage is typically progressive, and delayed diagnosis and treatment can lead to permanent vision loss. A case report described a patient with IgG4-related disease who experienced complete vision loss due to long-standing optic nerve damage (8), which improved to light perception after long-term glucocorticoid therapy. Another study (9) reported a 76-year-old female patient with IgG4-ROD who was misdiagnosed as having thyroid-associated ophthalmopathy (TAO) associated with hypothyroidism, accompanied by optic nerve compression by a mass. After two months of methylprednisolone pulse therapy, her best-corrected visual acuity (BCVA) and visual field defects improved significantly. This study suggests that IgG4-ROD can be masked by lesions associated with TAO. Both IgG4-ROD and TAO are immune-mediated inflammatory eye diseases. The key distinguishing features are that IgG4-ROD is characterized by lacrimal gland enlargement, IgG4-positive plasma cell infiltration, and systemic involvement of multiple organs, while TAO is characterized by proptosis, eyelid retraction, thyroid dysfunction, and thickening of the extraocular muscles. Given the patient’s elevated TSH levels, after consultation with an endocrinologist, subclinical hypothyroidism was considered. TAO can present with proptosis and is the most common cause of extraocular muscle hypertrophy. However, the patient tested negative for thyroid antibodies and did not exhibit typical symptoms of hyperthyroidism, such as palpitations or tremors. Additionally, TAO typically presents with symmetrical enlargement of the extraocular muscles, primarily the inferior rectus and medial rectus muscles. In this case, all four rectus muscles of the right eye were affected, and the involvement was unilateral, which does not align with the characteristics of TAO. Therefore, the diagnosis of TAO was ruled out.

IgG4-related disease (IgG4-ROD) and idiopathic orbital inflammatory pseudotumor (IOIP) are both inflammatory conditions of the orbit, and clinically, the two are difficult to distinguish. Studies have found that over 60% of biopsy-confirmed cases of IOIP are reclassified as IgG4-ROD, which has a stronger proliferative potential (10). IOIS refers to non-infectious, non-specific inflammatory reactions in the orbit, often presenting as unilateral, with symptoms such as proptosis, diplopia, eyelid redness and swelling, vision loss accompanied by significant orbital pain, and typically without systemic symptoms. Pathologically, it is characterized by infiltration of various inflammatory cells and varying degrees of fibrosis, with no specific laboratory markers. Studies indicate that the clinical course of IgG4-ROD is typically slow, whereas idiopathic orbital inflammatory pseudotumor often exhibits a more aggressive clinical course, leading to severe ocular dysfunction even after remission (11). Although this patient presented with proptosis, enlarged lacrimal glands, and thickened extraocular muscles, there were no significant ocular pain symptoms, only occasional ocular discomfort. Although no pathological biopsy was performed, the most critical distinguishing point was that the patient’s serum IgG4 levels were significantly elevated, thus temporarily ruling out idiopathic orbital inflammatory pseudotumor.

Some patients with IgG4-ROD may develop lymphoma, typically of the marginal zone mucosa-associated lymphoid tissue (MALT) type. IgG4-ROD may be a risk factor for the subsequent development of low-grade B-cell lymphoma (12). Recent studies indicate that in orbital lymphoid tissue diseases, approximately 55% of confirmed malignant tumors are orbital lymphomas, and nearly 50% of benign cases are IgG4-ROD (13). However, due to non-specific features, distinguishing between the two conditions is challenging. Lymphoma may be primary or part of systemic lymphoma, commonly affecting the lacrimal gland region, and may also involve the anterior orbital region and conjunctiva. The tumor may present as an arc-shaped mass encircling the eyeball or a cast-like lesion, and may simultaneously involve both the medial and lateral aspects of the orbital wall (14). IgG4-ROD and orbital lymphoma share highly similar clinical manifestations and imaging features. Studies have shown that orbital lymphoma is often unilateral and frequently involves the eyeball or optic nerve, which closely resembles the imaging characteristics of this case (13). Pathological diagnosis is the gold standard for differentiation between the two conditions. The pathological hallmark of lymphoma is monoclonal lymphocyte proliferation, without specific IgG4^+^ plasma cell infiltration. Although the patient in this case did not undergo pathological biopsy for confirmation, orbital lymphoma is often accompanied by fever, rapidly progressing masses, and lymphadenopathy in multiple sites. serum IgG4 levels are typically normal or mildly elevated, and imaging often shows infiltrative masses with bone destruction, necrosis, and hemorrhagic features, which do not align with this case, thus ruling out orbital lymphoma. A study using machine learning analysis found that miR-202-3p and miR-7112-3p are the optimal discriminating factors for IgG4-ROD and orbital MALT lymphoma, respectively (15). Additionally, a recent study conducted single-cell transcriptomics comparison analysis between MALT lymphoma of the ocular adnexa and IgG4-ROD, revealing that all B-cell subsets in MALT lymphoma are malignant, exhibiting significant intratumoral and intertumoral heterogeneity. CD4+ naive T cells in MALT lymphoma patients are highly likely to differentiate into follicular helper T cells, while in IgG4-ROD patients, CD4+ naive T cells are highly likely to differentiate into regulatory T cells (16). Proliferation-inducing ligand is highly expressed in lacrimal gland lesions of IgG4-ROD and MALT lymphoma patients. This overexpression may promote the enrichment of CD138(+) plasma cells and is associated with elevated serum IgG4 levels in IgG4-ROD patients (17). Additionally, it may promote the proliferation of CD20(+) B lymphocytes in MALT lymphoma patients. Proliferation-inducing ligand may play a role in the potential transformation of IgG4-ROD into MALT lymphoma.

Another critical differential diagnosis is ANCA-associated vasculitis (AAV), which can manifest with retro-orbital masses, sinusitis, and pulmonary nodules—features overlapping with IgG4-ROD. However, this patient had negative ANCA antibodies (not detected in serum), normal renal function tests (serum creatinine 68 μmol/L, estimated glomerular filtration rate 92 mL/min/1.73m²), and urinalysis showing no proteinuria or microhematuria, effectively excluding AAV-related renal or orbital involvement. Multicentric Castleman’s disease was also considered, as it may present with elevated serum IgG4 levels and systemic lymphadenopathy. However, the patient had no peripheral or mediastinal lymphadenopathy on chest CT, and Castleman’s disease typically lacks the orbital soft tissue masses and lacrimal gland enlargement observed here. Infectious etiologies such as tuberculosis were ruled out by negative tuberculin skin test (PPD) and T-SPOT.TB results, and the absence of constitutional symptoms (e.g., fever, night sweats, weight loss) further supported this exclusion. Other infections (e.g., fungal sinusitis) were unlikely given the lack of nasal discharge, normal inflammatory markers (CRP 8 mg/L), and absence of fungal elements in serum or imaging.

Mikulicz’s disease is a localized condition primarily affecting the salivary and lacrimal glands, characterized by bilateral chronic sclerosis of the lacrimal glands accompanied by swelling of the parotid and submandibular glands, along with elevated serum IgG4 levels. Recent studies suggest it is a subtype of IgG4-ROD, both of which are responsive to hormone therapy (18). However, IgG4-ROD is often associated with systemic involvement of multiple organs, such as maxillary sinusitis and ethmoid sinusitis, multiple pulmonary nodules, and hepatic calcifications in this case. Additionally, the patient does not exhibit symmetrical enlargement of the lacrimal or salivary glands, so Mikulicz’s disease is temporarily ruled out.

The patient in this case refused a pathological biopsy. The clinical diagnosis of IgG4-ROD was primarily based on the following criteria: (1) significantly elevated serum IgG4 levels; (2) orbital imaging showing a spindle-shaped mass surrounding the optic nerve, enlarged lacrimal glands, and thickening of the four extraocular muscles; (3) sensitivity to steroid therapy. However, the absence of histopathological confirmation remains a key limitation. Although the clinical, radiological, and serological findings strongly support the diagnosis of IgG4-ROD, a biopsy would have provided definitive evidence (e.g., IgG4-positive plasma cell infiltration and fibrosis). In clinical practice, biopsy should be prioritized when feasible to confirm the diagnosis and exclude other mimickers such as lymphoma or malignancies. Following steroid pulse therapy, the patient’s symptoms improved rapidly. Proptosis decreased from 23 mm to 19 mm, thickening of the extraocular muscles improved, the optic nerve mass significantly reduced in size, visual acuity improved from 0.1 to 0.25, and serum IgG4 levels decreased to 5.56 g/L, and further to 3.35 g/L one month later, confirming the accuracy of the diagnosis and avoiding the need for invasive biopsy. During subsequent outpatient follow-up, the patient’s vision remained stable with normal intraocular pressure and no significant protrusion of the right eyeball. Serial laboratory monitoring showed persistently normalized serum IgG4 levels: 3.65 g/L on June 13, 2025, and 3.31 g/L on August 18, 2025. Additional investigations during hospitalization, including tumor markers, ANA antibody profile, renal function tests, and urinalysis, were all within normal ranges, further excluding alternative diagnoses such as ANCA-associated vasculitis or lymphoma. A repeat enhanced orbital MRI is planned for the next follow-up to assess long-term anatomical resolution. Currently, there is controversy over whether to choose needle biopsy or surgical resection of the mass when surgical conditions are available. A retrospective analysis study included the biopsy group, which consisted of patients who underwent minimal tissue resection for diagnostic purposes, and the debulking group, which consisted of patients who underwent resection of most of the tumor to reduce its size. Recurrence rates were 71.4% and 12.5% in the biopsy and debulking groups, respectively, and 57.1% and 12.5% of patients required maintenance therapy with corticosteroids. Debulking surgery effectively reduced the need for postoperative steroid administration in IgG4-related disease patients with recurrent lacrimal gland lesions, suggesting the potential of surgical resection as an effective alternative to current standard treatment (19).

Of course, surgical treatment is not the only option. Drug therapy, especially glucocorticoids, remains an important first-line treatment for IgG4-ROD. Intravenous glucocorticoid therapy is well tolerated, provides better clinical remission, and is more effective than oral steroids in preventing inflammatory recurrence (20). Of course, there are differences in dosage regimens across regions, and further research is needed to establish standardized dosage guidelines. A recent study compared patients diagnosed with IgG4-ROD who initially received monotherapy with glucocorticoids, combination therapy with glucocorticoids and immunosuppressants (mycophenolate mofetil, azathioprine, hydroxychloroquine), monotherapy with biologics (rituximab), or observation and waiting. The primary outcome was treatment response assessed at 6 months, and the secondary outcome was assessment of recurrence 1 year after initial treatment. The study confirmed that combination therapy with glucocorticoids and immunosuppressants for IgG4-ROD can be considered an effective treatment method, and hydroxychloroquine can be regarded as a potential adjunctive therapy for IgG4-ROD (21). Additionally, obinutuzumab is a safe and promising treatment option for IgG4-ROD, rapidly reducing ocular inflammation and serum IgG4 levels (22). Studies have also shown that the IL-33/ST2/MMP-12 signaling pathway is activated in IgG4-ROD, IL-33/ST2 may promote M2 macrophage polarization and activation to produce MMP-12, which may serve as a novel therapeutic target for IgG4-ROD (23).

## Conclusion

IgG4-ROD is a chronic, relapsing immune-mediated condition that requires careful and long-term follow-up, along with comprehensive management of multisystem involvement. Clinically, the importance of multidisciplinary collaboration should be emphasized, including rheumatologists who can provide insights into immune-mediated pathogenesis and long-term immunosuppressive management (24). This case highlights that for patients presenting with unilateral proptosis and visual impairment, if serum IgG4 levels are significantly elevated and imaging demonstrates thickening of the rectus muscles plus soft tissue masses, IgG4-ROD should be strongly suspected. Misdiagnosis of IgG4-ROD may lead to inappropriate treatment, such as surgical removal of the mass, delaying steroid intervention, and progression of IgG4-ROD may result in optic nerve compression and irreversible vision loss. Early identification and steroid therapy can significantly improve symptoms, and procedures such as biopsy, surgical tumor reduction, immunosuppressive agents, and B-cell depletion therapy are being incorporated into standard treatment protocols. This case provides a typical reference for the diagnosis and treatment of clinically similar cases of optic nerve masses that are prone to misdiagnosis.

The bellies of the right medial rectus and lateral rectus muscles show symmetric thickening, presenting isointense signals on T1WI. Their tendon insertion sites are clear, and moderate homogeneous enhancement is seen on contrast-enhanced scan, with no “swelling at the myotendinous junction”. The right lacrimal gland displays diffuse enlargement, showing isointense signals on T1WI and homogeneous enhancement after contrast administration. C, D: T1WI coronal and sagittal images, respectively. The mass, thickened extraocular muscles, and lacrimal gland all present moderate homogeneous enhancement, with an enhancement degree lower than that of blood vessels. No “ring enhancement” or “necrotic area” is noted. The mucosa of the bilateral maxillary sinuses and ethmoid sinuses is thickened with mild enhancement.

## Data Availability

The raw data supporting the conclusions of this article will be made available by the authors, without undue reservation.
